# Inhibition of Alkali-Carbonate Reaction by Fly Ash and Metakaolin on Dolomitic Limestones

**DOI:** 10.3390/ma15103538

**Published:** 2022-05-15

**Authors:** Huan Cao, Zhongyang Mao, Xiaojun Huang, Min Deng

**Affiliations:** 1 College of Materials Science and Engineering, Nanjing Tech University, Nanjing 211800, China; 201961203093@njtech.edu.cn (H.C.); mzy@njtech.edu.cn (Z.M.); 5967@njtech.edu.cn (X.H.); 2 State Key Laboratory of Materials-Oriented Chemical Engineering, Nanjing 211800, China

**Keywords:** alkali-carbonate reaction, fly ash, metakaolin, concrete microbars, inhibition, expansion

## Abstract

In this paper, the dolomitic limestone determined as alkali–carbonate-reactive by various methods is used as an aggregate. Inhibition experiments were carried out on the basis of the concrete microbar method (RILEM AAR-5 standard), in which 10%, 30%, and 50% fly ash and metakaolin were used to replace cement. Thermogravimetric–differential scanning calorimetry (TG-DSC), X-ray diffractometry (XRD), mercury intrusion porosimetry (MIP), and scanning electron microscopy–energy dispersive X-ray spectrometry (SEM-EDS) were used to analyze the inhibition mechanism of fly ash and metakaolin on ACR. The results show that the expansion of samples at the age of 28 days are less than 0.10% when the fly ash contents exceed 30% and the metakaolin contents exceed 10%, which proves that the ACR is inhibited effectively. Meanwhile, the Ca(OH)_2_ content of the samples was reduced and the pore structure of the samples was optimized after adding fly ash and metakaolin. The dolomite crystals in the samples containing 50% fly ash and metakaolin are relatively complete.

## 1. Introduction

The alkali–aggregate reaction (AAR) includes the alkali–silica reaction (ASR) and the alkali–carbonate reaction (ACR), ACR is a reaction that occurs between the carbonate components of the aggregate and the alkaline solution, resulting in anomalous expansion and disintegration of the concrete. In 1957, Swenson [1,2] first discovered the case of engineering damage caused by ACR. At that time, he found that some concretes using carbonate aggregates in Kingston, Canada produced severe network cracks. As the microscopic characteristics of the cracks produced by these concretes were very different from those caused by ASR, he proposed that the carbonate component contained in the aggregate would react with the alkaline solution and cause cracking of the concrete structure. As a result, scholars from various countries have studied ACR, and a series of ACR expansion mechanisms have been proposed.

Since the ACR was proposed, there has been a relatively consistent view [3]. The ACR is a chemical reaction between the alkali (K^+^, Na^+^) in the pore solution and the dolomite (CaMg(CO_3_)_2_) in the aggregate (also known as a dedolomization reaction), and the reaction equation is as follows:

CaMg(CO_3_)_2_ + 2MOH → Mg(OH)_2_ + CaCO_3_ + M_2_CO_3_ (1)

where M represents alkali metal ions: K^+^, Na^+^, or Li^+^

M_2_CO_3_ + Ca(OH)_2_ → 2MOH + CaCO_3_, (2)

Reaction Formula (1) is the dedolomitization reaction; Formula (2) indicates that M_2_CO_3_ continues to react with Ca(OH)_2_ to form calcite and regenerate MOH.

Measures commonly adopted to mitigating AAR include selective quarrying of aggregates, use of low-alkali cements, incorporating chemical admixtures or mineral admixtures such as pulverized fly ashes and granulated blast furnace slag, and limiting the alkali content of concrete. Unfortunately, nonreactive aggregates are not always supplied. Other measures are necessarily recommended to mitigate expansion caused by AAR.

Mineral admixtures such as silica fume, pulverized fly ash, and granulated blast furnace slag are widely used to inhibit ASR expansion [4,5,6]. The effectiveness of mineral admixtures in controlling ASR is generally derived from decreasing of content of cementitious materials, lowering OH- concentrations in pore solutions by adsorption of low Ca/Si C–S–H gels and resisting migration of ions in pore solutions of concrete by fining pores [7].

Mineral admixtures are not as effective in controlling ACR as it is to conduct an ASR when Portland cement with 0.43% Na_2_O equivalent is used [8,9,10,11]. Replacement of cements with pulverized fly ash (PFA), 90% blast-furnace slag (BFS), or 30% silica fume (SF) is needed to effectively inhibit ACR expansion of reactive dolomitic limestones from Kingston, Canada. It seems impossible to inhibit deleterious ACR in the Portland cement system in practice because the necessary content of mineral admixtures is too high [9,12].

Granulated blast furnace slags (GBFS) lend to reduce the hydroxyl ion concentration in pore solutions and restrict the transportation of water and ionic species to reaction sites when blended with Portland cement [8]. It was found that activated blast furnace slag cement (ABFSC) concretes are found to be more susceptible to ACR expansion. Sulfoaluminate and supersulfated cements were thought to be effective in inhibiting ACR expansion because the pH value of their pore solutions is about 9.43–10.82 and 12.00, respectively [4].

FeCl_3_ and Li_2_CO_3_ were chosen as admixtures to control the expansion of concrete made with the highly reactive dolomitic limestones from Kingston and a high-alkali Portland cement. The expansion of the concrete was reduced by 50% and the effectiveness of the additives in mitigating expansion was not enough to prevent the concrete from cracking [8,13]. The reduction of OH^-^ concentrations in the pore solutions due to the formation of Fe(OH)_3_ may be involved in the reducing the expansion of the concrete.

In research on the chemical resistance of concrete, it was noted that newly formed magnesium hydroxide can react with silica gel to form magnesium silicate hydrate, which tended to block pores and hindered penetration of ions [14]. The experiment was conducted to investigate the expansion of reactive carbonate rock prisms and concrete microbars in alkaline solution with silica gel. Rock prisms were cut from three alkali-reactive rocks from Kingston, Canada. Concrete microbars were prepared according to the method proposed by Tang [15]. The pH value of curing solution was kept at 13.4. The results suggested that silica gel is beneficial to inhibiting the expansion resulting from ACR [4].

It is generally accepted that low-alkali Portland cements, mineral admixtures, and lithium salts cannot be used to effectively mitigate the expansion of highly reactive dolomitic limestones from Kingston, Canada. Neither may limiting alkali content of concrete effectively control the expansion of highly reactive dolomitic limestones from Kingston, Canada. Data on the effectiveness of measures on preventing ACR are almost completely based on the highly reactive dolomitic limestones from Kingston, Canada. Not enough data can be obtained on preventing the expansion of moderately reactive dolomitic rocks. Nowadays, alkali–carbonate reactive rocks are not allowed to be used as coarse aggregates of concrete in specifications globally.

The main purpose of this study is to examine the inhibition and mechanism of fly ash and metakaolin on the ACR. Therefore, in this paper, concrete microbars were prepared by using alkali–carbonate reactive dolomitic limestones as aggregate, and fly ash and metakaolin were used to inhibit the ACR of dolomitic limestones. Thermogravimetric–differential scanning calorimetry (TG-DSC), X-ray diffractometry (XRD), mercury intrusion porosimetry (MIP), and scanning electron microscopy–energy dispersive X-ray spectrometry (SEM-EDS) were used to analyze the inhibition mechanism of fly ash and metakaolin on ACR. The study will guide the practical application of dolomite in concrete structures.

## 2. Materials and Methods

### 2.1. Materials

#### 2.1.1. Cementitious Materials

P·II 52.5 Portland cement (OPC) from Jiangnan—Onada Cement Co. Ltd. Nanjing, China was used in this experiment. Its alkali content was 0.56%. The chemical composition of the cement was analyzed by X-ray fluorescence (XRF) in Table 1.

**Figure 1:**
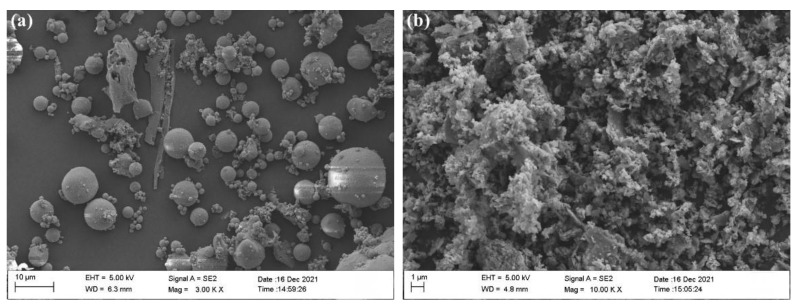
SEM image of (**a**) fly ash and (**b**) metakaolin.

Metakaolin (MK) is produced by Super Kaolin Co. Ltd. Inner Mongolia, China. It is calcined and ground from kaolin ore. Table 1 shows its chemical composition. MK is mainly in the form of amorphous aluminum silicate. The morphology of metakaolin observed by scanning electron microscope (SEM) is shown in Figure 1.

Class F fly ash (FA) from Nanjing Pudi, Table 1 shows its chemical composition. The particle morphology of fly ash observed by scanning electron microscope (SEM) is shown in Figure 1.

#### 2.1.2. Aggregates

**Figure 2:**
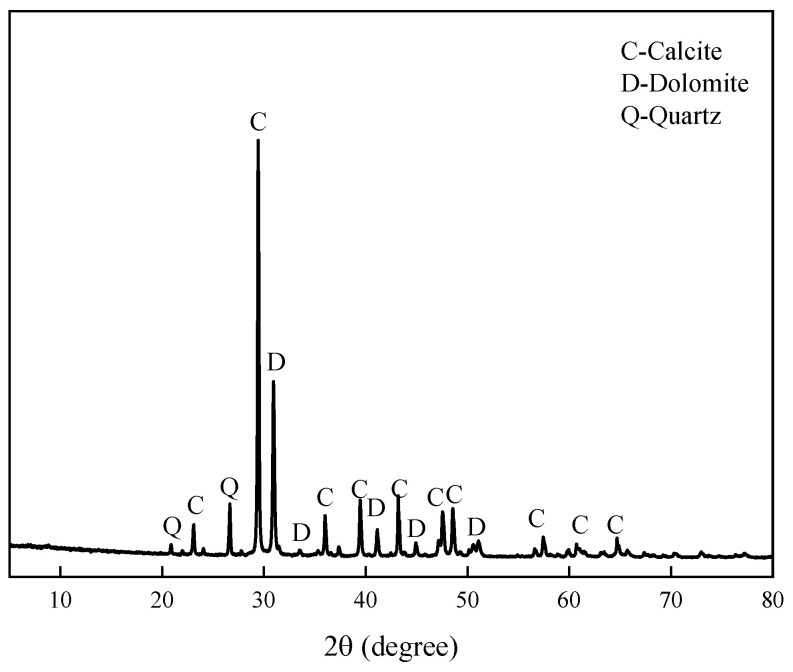
XRD patterns of dolomitic limestone S1# from Baofuling, Shandong, China.

**Figure 3:**
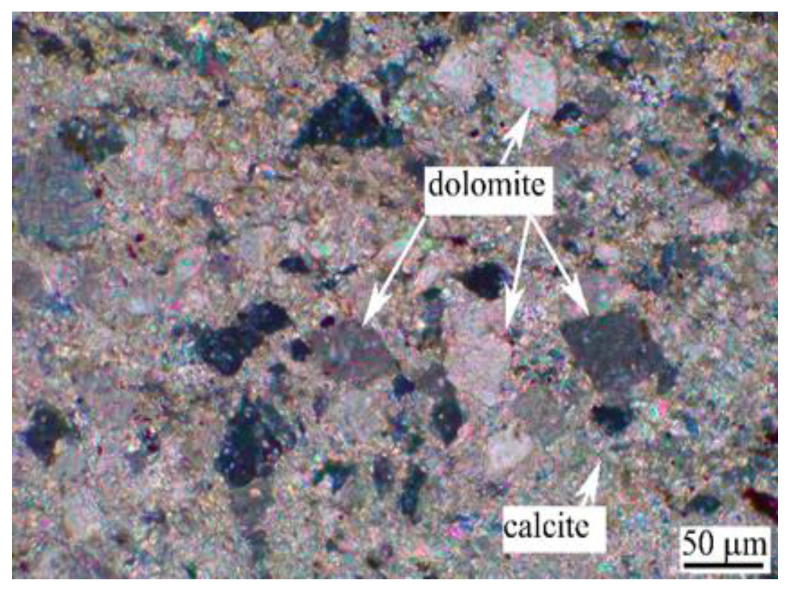
Petrographic micrographs of dolomitic limestone S1# from Baofuling, China.

The aggregate is Ordovician dolomitic limestone S1# derived from Baofuling, Shandong Province, China. Table 2 shows its chemical composition. The XRD patterns of dolomitic limestone S1# are demonstrated in Figure 2. The dolomitic limestone S1# is mainly composed of calcite, dolomite, and quartz. The typical microstructure of dolomitic limestones by is shown in Figure 3; dolomite crystals in dolomitic limestone S1# are dispersed in the calcite matrix.

### 2.2. Methods

#### 2.2.1. Determination of Aggregate Alkali Activity

The concrete microbars method (RILEM AAR-5) [16], accelerated mortar bars method (ASTM C1260) [17], rock prisms method (ASTM C586) [18], and concrete prisms method (ASTM C1293) [19] were used to determine the alkali activity of carbonate aggregate S1#. Inhibition experiments were performed on the basis of the concrete microbars method (RILEM AAR-5).

#### 2.2.2. Concrete Microbars Test

According to the standard of RIELM AAR-5, the experiment used dolomitic limestone S1# as aggregate (around 5 mm–10 mm) and Portland cement (alkaline content adjusted to 1.25% with NaOH) to prepare concrete microbars (40 mm × 40 mm × 160 mm), the cement to aggregate ratio is 1:1, and the water–cement ratio is 0.32. Fly ash and metakaolin replaced 10%, 30%, and 50% of cement quality respectively. Table 3 shows a mixed design of concrete microbars. The concrete microbars were cured in a solution of 1M NaOH at 80 °C, and the expansion of the concrete microbars were measured regularly. If the expansion of the concrete microbars at age of 28 days is less than 0.1%, it is considered that ACR can be inhibited.

#### 2.2.3. Determination of Hydration Products

Thermogravimetric–differential scanning calorimetry (TG-DSC) and X-ray diffractometry (XRD) were used to determine the change of Ca(OH)_2_ content in the concrete.

Cement pastes containing 10%, 30%, and 50% fly ash and metakaolin were made into about 2–3 mm cubes. The TG-DSC test used the STA 449 F3 thermal analyzer from NETCSZH Company, Bayern, Germany. Samples were heated from 0 to 600 °C at a heating rate of 10 °C/min in a N_2_ atmosphere, and α-Al_2_O_3_ was used as the reference to analyze the heat and mass changes of the samples during the heating process.

The cement pastes containing 10%, 30%, and 50% fly ash and metakaolin were dried, ground with an agate mortar, the particle size was controlled below 80 μm, and the samples filled into a 20 × 20 × 0.5 mm quartz sample tank. The XRD test used the SmartLab (3) X-ray diffractometry (Cu target, rated power 3 kW, scanning range of 5°–80° with a step size 0.02° and scanning speed of 10°/min) from Rigaku Company, Tokyo, Japan.

#### 2.2.4. Mercury Porosimetry Analyses

A cube about 2–3 mm in the center of the concrete microbars containing 10%, 30%, and 50% fly ash and metakaolin was cut and PoreMaster-60 mercury porosimeter was used to analyze the pore structure of the selected samples. The test has two phases—low pressure from 0 psi to 25 psi (0.172 MPa) and high pressure from 20 psi (0.138 MPa) to 50,000 psi (344.738 MPa).

#### 2.2.5. Microscopic Analysis of ACR Products

Ultra55 field emission scanning electron microscopy from Zeiss Company Jena, Germany was used to observe the morphology of the ACR products in concrete microbars.

## 3. Results and Discussion

### 3.1. Alkali Activity of Aggregate

Table 4 shows the expansion of accelerated mortar bars (ASTM C1260), concrete microbars (RILEM AAR-5), concrete prisms (ASTM C1293), and rock prisms (ASTM C586). The accelerated mortar bars method was used to determine the alkali–silica reactivity of dolomitic limestones—if the expansion of the mortar bars at the age of 14 days exceeds 0.1%, the aggregates are classified as potentially alkali–silica-reactive. The concrete microbars method, concrete prisms method, and rock prisms were used to determine the alkali–-carbonate reactivity of dolomitic limestones. If the expansion of the concrete microbars at age of age of 28 days exceeds 0.10%, the aggregates are classified as alkali–carbonate-reactive. If the expansion of the concrete prisms at the age of 1 year exceeds 0.04%, the aggregates are classified as alkali–carbonate-reactive. If the expansion of the rock prisms at age of 84 days exceeds 0.10%, the aggregates are classified as alkali–carbonate-reactive. Four methods were used to prove that aggregate S1# were classified as alkali–carbonate-reactive and not alkali–silica-reactive.

### 3.2. Inhibition of ACR with Fly Ash

**Figure 4:**
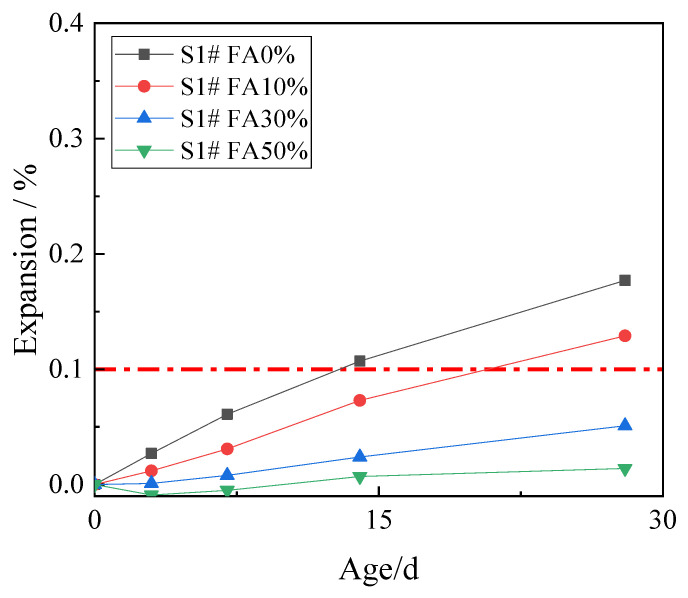
The expansion of the concrete microbars with fly ash.

In order to study the effect of fly ash on inhibiting ACR, 10%, 30%, and 50% fly ash was used to replace cement. The expansion rates of concrete microbars at the age of 28 days are shown in Figure 4.

Figure 4 shows that the expansion of the control samples continued to develop with age, and the expansion at age of 28 days was 0.177%. The fly ash reduced the ACR expansion of the samples, and the higher the fly ash content, the smaller the expansion. The expansion of the concrete microbars containing 10% fly ash at age of 28 days was 0.129% which is still higher than the defined threshold (0.1%), which indicates that the low proportion of fly ash has poor ACR inhibition effect on the samples. When the content of fly ash increased to 30% and 50%, the expansion of concrete microbars at age of 28 days decreased to 0.051% and 0.014%. The ACR expansion of the samples was significantly inhibited. The results show that only the fly ash with high mass fractions can significantly inhibit the ACR of the samples, while the fly ash with low mass fraction cannot inhibit the ACR of the samples effectively.

### 3.3. Inhibition of ACR with Metakaolin

**Figure 5:**
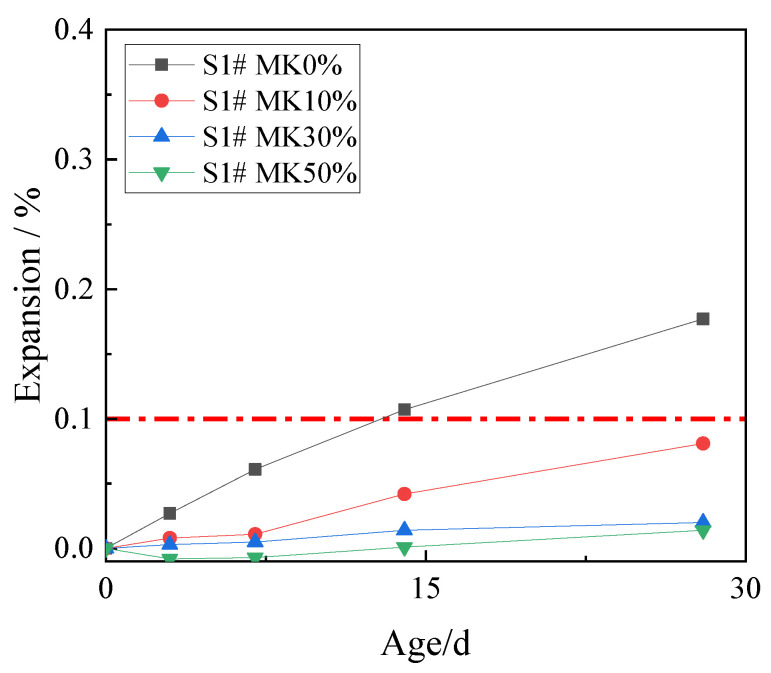
The expansion of the concrete microbars with metakaolin.

In order to study the effect of metakaolin on inhibiting ACR, 10%, 30%, and 50% metakaolin was used to replace cement. The expansion rates of concrete microbars at the age of 28 days are shown in Figure 5.

Figure 5 shows that metakaolin has an excellent inhibitory effect on ACR. The expansion of concrete microbars at age of 28 days with metakaolin contents of 10%, 30%, and 50% were 0.081%, 0.020%, and 0.007%, which were all lower than the defined threshold (0.1%). Compared with the control samples, 10% metakaolin can effectively inhibit the ACR of the samples. The inhibitory effect of 30% and 50% metakaolin is more obvious. The results show that metakaolin is highly effective in inhibiting ACR.

### 3.4. Effects of Fly Ash and Metakaolin on Ca(OH)_2_ in Cement Paste

As one of the important hydration products of cement, Ca(OH)_2_ has been recognized by many scholars for its role in maintaining the alkalinity of the cement system and promoting AAR.

#### 3.4.1. XRD Analyses

**Figure 6:**
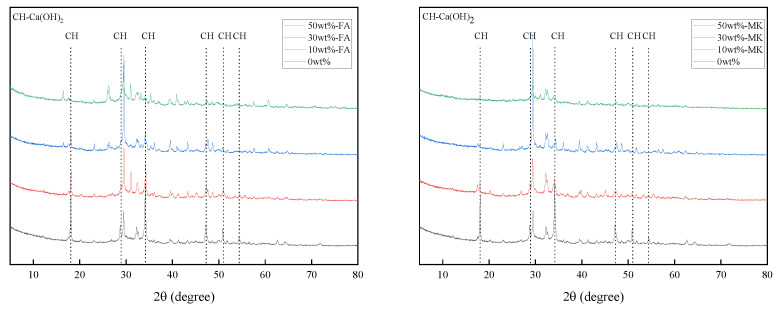
XRD patterns of cement pastes containing 0%, 10%, 30%, and 50% fly ash and metakaolin that were cured at 20 °C for 28 days.

X-ray diffraction analysis can semi-quantify the change of Ca(OH)_2_ content in cement paste. Figure 6 are XRD patterns of cement pastes containing 0%, 10%, 30%, and 50% fly ash and metakaolin that were cured at 20 °C for 28 days. With the increase of the content of fly ash and metakaolin, the diffraction peak intensity of Ca(OH)_2_ in the XRD pattern also decreases, which indicates that the content of Ca(OH)_2_ in the cement paste decreases with the increase of the content of fly ash and metakaolin.

#### 3.4.2. TG-DSC Analyses

TG-DSC can accurately determine the content of Ca(OH)_2_ in cement paste, which can be accurate to less than 0.1%.

**Figure 7:**
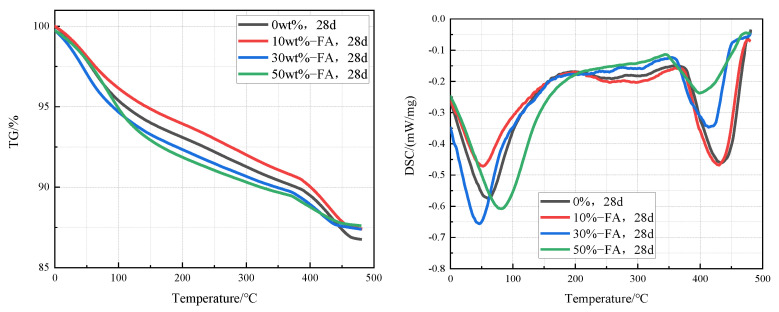
TG-DSC curves of cement pastes containing 0%, 10%, 30%, and 50% fly ash after curing at 20 °C for 28 days.

Figure 7 is TG-DSC curves of cement pastes containing 0%, 10%, 30%, and 50% fly ash that were cured in an aqueous solution at 20 °C for 28 days. It can be clearly found from the curves that the intensity of the Ca(OH)_2_ endothermic peak in the cement pastes decreases significantly with the increase of the fly ash content, which indicates that the content of Ca(OH)_2_ in the cement paste decreases with the increase of fly ash content. According to the TG-DSC curve, the content of Ca(OH)_2_ in cement paste can be calculated. The contents of Ca(OH)_2_ in cement paste containing 0%, 10%, 30%, and 50% fly ash were 12.3%, 11.5%, 9.0%, and 7.4% respectively.

**Figure 8:**
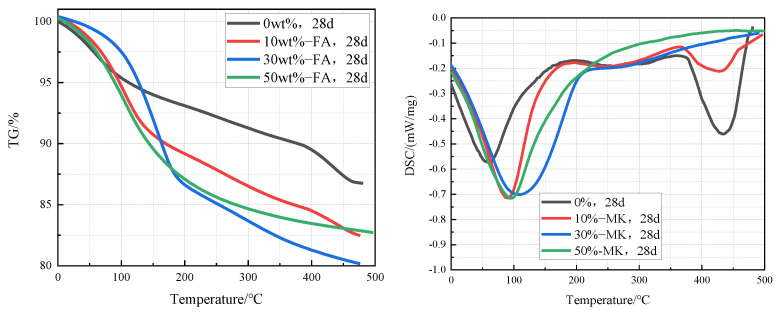
TG-DSC curves of cement pastes containing 0%, 10%, 30%, and 50% metakaolin after curing at 20 °C for 28 days.

Figure 8 is TG-DSC curves of cement pastes containing 0%, 10%, 30%, and 50% metakaolin that were cured in an aqueous solution at 20 °C for 28 days. It can be clearly seen from the curves that the intensity of the Ca(OH)_2_ endothermic peak in the cement pastes decreases significantly with the increase of the fly ash content, which indicates that the content of Ca(OH)_2_ in the cement paste decreases with the increase of fly ash content. According to the TG-DSC curve, the content of Ca(OH)_2_ in the sample can be calculated. The contents of Ca(OH)_2_ in cement paste containing 0%, 10%, 30%, and 50% fly ash were 12.3%, 8.2%, 0%, and 0% respectively, which indicates that the Ca(OH)_2_ in cement pastes containing 30% and 50% metakaolin has been completely reacted.

There are two main reasons for this result. On the one hand, the content of cement decreased after the fly ash and metakaolin partially replaced the cement, and the content of Ca(OH)_2_ generated by cement hydration also decreased. On the other hand, the secondary pozzolanic reaction of Ca(OH)_2_ with fly ash and metakaolin resulted in the decrease of Ca(OH)_2_ content in the system. With the hydration of cement, the fly ash and metakaolin react with hydration products Ca(OH)_2_ and C-S-H gel for secondary pozzolanic reaction, further producing C-S-H gel with low Ca/Si. The reduction of the content of Ca(OH)_2_ weakens the regeneration of alkali metal ions (Na^+^, K^+^) and the secondary pozzolanic reaction produces a large amount of C-S-H gel with low Ca/Si, which has a stronger adsorption effect on alkali metal ions (Na^+^, K^+^).

### 3.5. Effects of Fly Ash and Metakaolin on the Pore Structure of Concrete Microbars

**Figure 9:**
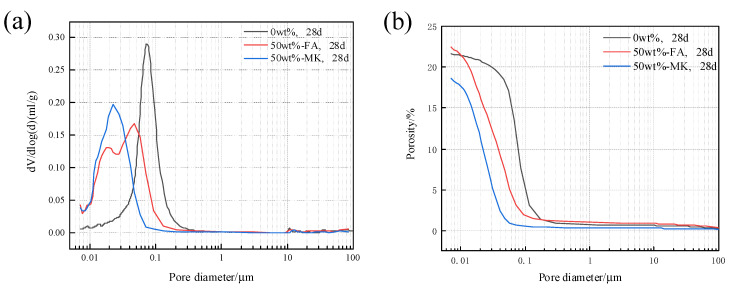
Pore structure of samples cured in 1 M NaOH at 80 °C for 28 days: (**a**) pore size distribution, (**b**) cumulative porosity.

The pore structure of concrete microbars cured in 80 °C, 1M NaOH solution for 28 days was analyzed. Figure 9 shows the pore size distribution and cumulative porosity of concrete microbars containing 50% fly ash and metakaolin. The porosity of the blank sample was 21.64%, and the porosity of the sample containing 50% fly ash reached 22.41%. The porosity of the samples containing fly ash increased; however, the pore size distribution tends to be smaller. The porosity of the samples containing 50% metakaolin is 18.60%, and the porosity of the samples is reduced and the pore structure is more compact. The physical filling effect of fly ash and metakaolin and the filling effect of secondary pozzolanic reactants reduce the porosity of the cement stone, reduce the pore size, and densify the structure, which may be beneficial to prevent the diffusion of K^+^ and Na^+^ to the active aggregate and inhibit ACR.

### 3.6. Effect of Fly Ash and Metakaolin on Aggregate Reaction Degree

**Figure 10:**
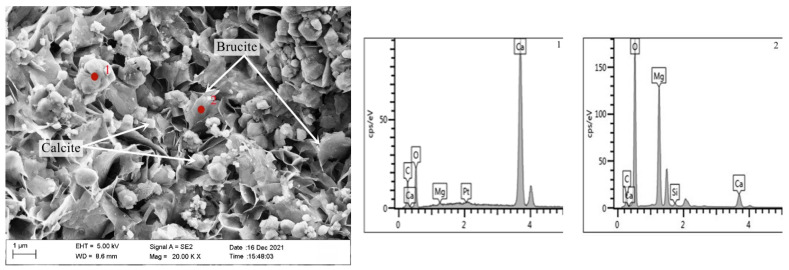
SEM-EDS image of the product in fracture surface of aggregate S1# in the concrete microbars cured in 1M NaOH solution at 80 °C for 28 days.

Figure 10 is a SEM-EDS diagram of the inner products of the concrete microbars prepared by rock S1# after curing for 28 days at 80 °C in 1M NaOH. As can be seen, a large number of columnar (point 1) and lamellar (point 2) products are formed. EDS results show that the columnar products are mainly composed of Ca, C, and O, which is calcite. The lamellar products are mainly composed of Mg and O, which is brucite—this indicates that the dedolomization reaction also took place in the rock prisms. The length and width of the brucite are about 1 μm and the calcite particles are about 0.5 μm. The brucite and calcite stack each other, and there are a lot of pores between the products. The crystal size of brucite and calcite is larger, and the flaky brucite and calcite stacked together are not dense, and there are obvious pores. It can be seen that the reaction product gradually grows up and more and more brucite and calcite gather together in a limited space with the progress of the reaction. At the same time, the accumulation of products leads to the increase of pores between products, which causes the continuous expansion of the rock, which is also the reason for the expansion of concrete microbars. The results show that the expansion of concrete microbars cured in NaOH solution is caused by a dedolomization reaction.

**Figure 11:**
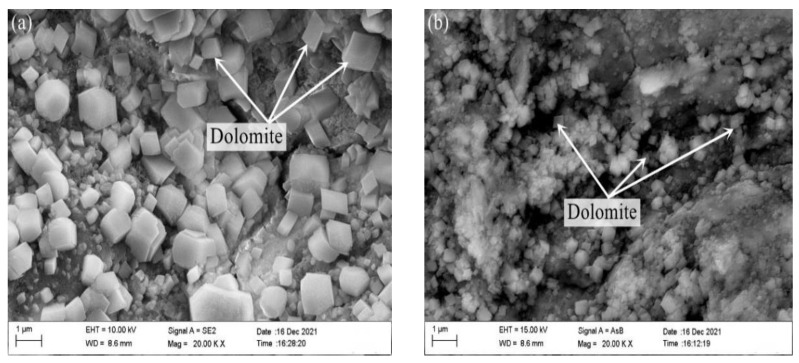
SEM image of the product in fracture surface of aggregate S1# in the concrete microbars cured in 1M NaOH solution at 80 °C for 28 days: (**a**) 50% metakaolin. (**b**) 50% fly ash.

Figure 11 is a SEM-EDS diagram of the inner products of the concrete microbars containing 50% fly ash and metakaolin prepared by rock S1# after curing for 28 days at 80 °C in 1M NaOH. It can be seen from the figure that the dolomite crystals inside the rock are relatively complete, and no lamellar brucite and columnar calcite generated by the dedolomization reaction were found, which indicates that the addition of 50% fly ash and 50% metakaolin inhibit the progress of the dedolomization reaction.

### 3.7. Effects of Fly Ash and Metakaolin on Expansion Cracks

**Figure 12:**
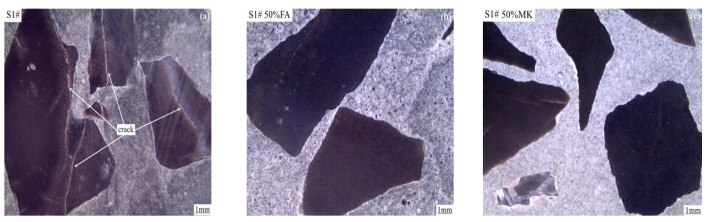
The expansion cracks of concrete microbars prepared with rock S1# cured in 1M NaOH solution at 80 °C for 56 days. (**a**) control group; (**b**) 50% fly ash; (**c**) 50% metakaolin.

Figure 12 shows the expansion cracks of concrete microbars prepared with rock S1# cured in 1M NaOH solution at 80 °C for 56 days. As can be seen from Figure 12a, obvious cracks appeared inside the concrete microbars without fly ash and metakaolin, and these cracks were mainly concentrated in the rock aggregate, from the inside of the rock to the the cement pastes extended, and no ASR gel was found near the aggregate, which indicated that the expansion cracking of the concrete microbars was caused by the ACR. It can be seen from Figure 12b,c that the cracks inside the concrete microbars containing 50% fly ash and 50% metakaolin almost completely disappeared. This indicates that the reduction of expansion cracks of concrete microbars prepared with rock S1# is directly related to the addition of fly ash and metakaolin.

## 4. Conclusions

The most significant conclusions of this paper are summarized as follows:

(1) Four methods were used to determine that the dolomitic limestone S1# is alkali-carbonate reactive aggregate.
(2) In the concrete microbars test, metakaolin and fly ash can effectively inhibit the ACR of reactive aggregate, and the more the content of fly ash and metakaolin, the more efficient the effect of inhibition. Less fly ash cannot effectively inhibit the alkali–aggregate reaction of active aggregate. When the content of fly ash and metakaolin is the same, metakaolin is more effective than fly ash.
(3) The products of ACR calcite and brucite can be clearly observed in the SEM image, but no ASR gel was found, which indicates that the expansion of dolomitic limestone S1# was caused by ACR.
(4) Metakaolin and fly ash participate in the secondary hydration of cement, which consumes a large amount of the hydration product Ca(OH)_2_ of Portland cement and reduces the alkalinity of the cement hydration product, so the ACR be inhibited. The pore structure of concrete containing fly ash and metakaolin becomes denser, which prevents the diffusion of K^+^ and Na^+^ to the active aggregate.

## Figures and Tables

**Table 1:** Chemical composition of cementitious materials (wt%).

Materials	CaO	MgO	SiO_2_	Al_2_O_3_	Fe_2_O_3_	K_2_O	Na_2_O	SO_3_	LO I	Totals
Cement	64.00	2.35	19.43	4.73	2.96	0.47	0.26	2.58	2.81	99.59
Fly ash	4.40	1.11	50.10	29.77	8.95	0.89	0.39	1.15	1.81	98.57
Metakaolin	0.37	0.43	52.00	43.00	1.10	0.40	0.30	0.14	0.50	98.24

**Table 2:** Chemical composition of dolomitic limestone S1# (wt%).

Sample	Chemical Composition/%					
	Loss	SiO_2_	Fe_2_O_3_	Al_2_O_3_	CaO	MgO
S1#	41.58	3.16	0.26	0.93	44.61	4.39

**Table 3:** Mix design of concrete microbars.

Sample Name	Cement/g	Fly Ash/g	Metakaolin/g	Aggregates/g	w/c
S1#0%	900	-	-	900	0.32
S1#FA10%	810	90	-	900	
S1#FA30%	630	270	-	900	
S1#FA50%	450	450		900	
S1#MK10%	810	-	90	900	
S1#MK30%	630	-	270	900	
S1#MK50%	450	-	450	900	

**Table 4:** Alkali-reactivities of dolomitic limestone S1# based on results of ASTM C1260, RILEM AAR-5, ASTM C1293, and ASTM C586.

Sample	Expansion of Mortar Bars at Age of 14 Days	Expansion of Concrete Microbars at Age of 28 Days	Expansion of Concrete Prisms at Age of 1 Year	Expansion of Rock Prisms at Age of 84 Days	Reactivity
S1#	0.087%	0.177%	0.0427%	0.114%	ACR

## Data Availability

The data presented in this study are available on request from the corresponding author.
